# Intent to share individual participant data of Indian clinical trials

**DOI:** 10.1016/j.lansea.2023.100318

**Published:** 2023-11-11

**Authors:** Ronak Borana, Jyoti Tyagi, Gian Luca Di Tanna, Vivekanand Jha, Soumyadeep Bhaumik

**Affiliations:** a Meta-research and Evidence Synthesis Unit, The George Institute for Global Health, India; b Department of Business Economics, Health and Social Care, University of Applied Sciences and Arts of Southern Switzerland, Switzerland; c The George Institute for Global Health, UNSW, India; d School of Public Health, Imperial College London, London, UK; e Prasanna School of Public Health, Manipal Academy of Higher Education, Manipal, India; f Meta-research and Evidence Synthesis Unit, The George Institute for Global Health, UNSW, Australia

Modern medicine and public health are based on the bedrock of evidence that tells us “what works”? The evidence-informed medicine movement has made the distinction between modern medicine and other systems of medicine stark. The science of systematic reviews and meta-analysis, which inform decisions on which therapies to use (or not), has evolved significantly in the last two decades. A key advance in this regard is the ability to conduct meta-analysis of Individual Participant Data (IPD).

IPD level meta-analysis, compared to traditional meta-analysis (which pools aggregate level statistics), enables proper assessment of intervention/treatment and covariates interactions by minimizing the risk of aggregate data bias. Availability of IPD level data also enables secondary analysis and independent re-analysis of trial data.^1^ The former allows more realistic estimates, while the latter enables detection of research fraud and inadvertent errors, thereby improving transparency and public trust in clinical trials and evidence-informed medicine. The availability of IPD from trials also mitigates the risk of outcome reporting bias. IPD may provide data on more outcomes than those reported in individual publications. Sharing IPD increases trust in the research conducted and may lead to higher citations, and further scientific collaborations. A recent study found a 25% increase^2^ in citations for studies that planned to share data. The ability to conduct IPD meta-analysis or meta-research (secondary analysis or re-analysis of trial data) depends on the availability of raw individual-level data from trials. There have been growing calls^3^^,^^4^ for clinical trials to support data sharing so that the information from trials can be used to its fullest extent.

Evidence synthesis specialists and those conducting meta-research on trials need to know which trials are open to share IPD. Clinical trial registries are an essential data source on trialists' intentions to share IPD. In 2019, the World Health Organization (WHO) made it mandatory to include a data field on IPD sharing plans in all primary clinical trial registries.^5^ An audit^6^ of 18 primary registries enlisted by the WHO found that only four registries globally, including the Clinical Trials Registry of India (CTRI), had IPD sharing field compliant with ICMJE's directive^4^ that mandated the mention of the IPD sharing plan in the trial registration for studies conducted after 2019.

India is a major hub of clinical trials globally, and the mandatory requirement of the IPD field in CTRI registry is a progressive move to support open data. However, it is unclear whether Indian trialists are willing to share IPD with evidence synthesis specialists or meta-researchers.

Our analysis of CTRI records from 2019 (See Appendix and interactive living dashboard here) shows that, overall, only about 12% of trialists conducting trials in India intended to share IPD. There has been a year-on-year progress. Starting from <1% in 2019, the intent increased to 12.6% in 2022. However, among trialists who committed to share, only 38% mentioned they would share all IPD data collected during the trial after de-identification. Trials that were industry-funded, multi-country, or required regulatory approval were less likely to commit to IPD data sharing, which seem to contrast the result of a global analysis (not including data from India)^7^ which reported a non-statistically significant difference between commercial and non-commercially funded trials.

Through this correspondence, we call for policy action on the part of all stakeholders to improve availability of IPD. Government, philanthropic and non-commercial research funders should add clauses in funding agreements to enhance IPD sharing. Regulatory agencies should mandate IPD sharing in industry-funded trials. Such mandates should specify timelines in relation to completion, early termination, or abandonment of trials.

There is also a need for awareness and capacity building about IPD among stakeholders. Research funders should invest in data management capacities in clinical trial centres to ensure that IPD data can be adequately prepared for public sharing. Improving awareness among ethics committees to recommend IPD sharing, with de-adequate safeguards is another potential strategy to consider. Development of strategies should be informed by qualitative studies on facilitators and barriers for IPD data sharing in India. The Indian Clinical Trial & Education Network (INTENT) of the Indian Council of Medical Research (ICMR), which is mandated to promote clinical trials relevant to the Indian context, can play an important role. The Findability, Accessibility, Interoperability, and Reuse (FAIR) data-sharing principles,^8^ which not only promotes data sharing but also provides measurable data principles that can serve as a guideline for navigating data sharing, should be adapted for the Indian context. The framework helps improve the visibility and impact of research and helps make the research more reproducible and reliable. Finally, the non-availability of IPD from Indian trials also slows down building the evidence base around various interventions for Indians.

In conclusion, IPD data sharing is a public good and will enhance the stature of Indian trials globally by ensuring transparency. We call for all stakeholders in India to work towards operationalising the steps needed to achieve this goal.

## Contributors

RB and SB wrote the first draft of this comment and contributed to the conception and design of the study, which provides background to this comment. All authors contributed to the interpretation of data. All authors critically reviewed the manuscript for significant intellectual content.

## Declaration of interests

None of the authors have competing interests.

The views expressed in this article are those of the authors and do not necessarily reflect the positions of their institution or their other funders.
