# Supplementary material for: Intent to share individual participant data of Indian clinical trials
Source: Lancet Reg Health Southeast Asia. 2023 Nov 11;19:100318. doi: 10.1016/j.lansea.2023.100318 (PMC10665899; doi:10.1016/j.lansea.2023.100318)
Supplement: Appendix [file mmc1.docx]

Appendix

# Brief Background

In February 2022, we conducted an audit ^1^ of the IPD data sharing field in 18 primary registries enlisted by the WHO. We found that registries from the Australian New Zealand Clinical Trials Registry (ANZCTR), ClinicalTrials.gov, Clinical Trials Registry - India (CTRI), and Iranian Registry of Clinical Trials (IRCT) had IPD sharing fields compliant with ICMJE's directive^2^ that mandated the mention the IPD sharing plan in the trial registration for studies conducted after 2019. While there is an analysis of trialists' intent to share IPD for the other three registries,^3,4,5^ to our knowledge, there is none for trials registered in CTRI. We aimed to fill this gap through the current study by analysing the intent of individual participant data sharing in records from CTRI after 2019 WHO mandate.

# Objectives

Our specific objectives are:

1. to assess the yearly prevalence of intent to share IPD; and
2. to analyse the factors associated with sharing data for IPD.

# Method

We included CTRI records from 1 January 2019 to 15 November 2022 to align with the WHO mandate. We excluded observational and bioavailability-bioequivalence trials and only included interventional & post-marketing surveillance trials.

We identified CTRI records from WHO International Clinical Trials Registry Platform (ICTRP)^6^. It lists all CTRI records, but the WHO ICTRP data does not include some fields related to the IPD data-sharing plan available in CTRI. We, therefore, used the web links from the ICTRP to download the rest of the data from the CTRI website.

To analyse trials based on their size, we arranged the trials in the ascending order of sample size and divided them into four quartiles.

For each record, we analysed the following trial characteristics: year of registration, sample size, type of sponsor, part of a postgraduate thesis, number of countries, regulatory approval, phase, and study design.

All the trials characteristics like ‘Type of Sponsor’, ‘Study Design’, ‘Regulatory Approval’ are defined by CTRI in detail in a dataset description document available on their official website (<https://ctri.nic.in/Clinicaltrials/CTRI_Dataset_and_Description.pdf>)

To enable visualization that can inform decision-makers and advocates working on the issue, we developed a live dashboard that will be updated at regular intervals.

# Results

We downloaded all 30,346 studies registered on CTRI between 1 January 2019 and 15 November 2022, and identified 21,910 trial records (21,563 interventional and 347 post-market surveillance trials) that met the criteria for the current analysis. Table 1 shows the basic characteristics of the included trials.

**Table 1 Study characteristics and the IPD sharing statement of CTRI records included in this analysis**

| **Fields** |  | **Not Available** | **No** | **Yes** |
| --- | --- | --- | --- | --- |
| Year | 2019-2022 (All) | 7173 (32ˑ74%) | 13032 (59ˑ48%) | 1705 (7ˑ78%) |
|  | 2019 | 3945 (93ˑ88%) | 226 (05ˑ38%) | 31 (0ˑ74%) |
|  | 2020 | 3196 (60ˑ31%) | 2052 (38ˑ72%) | 51 (0ˑ96%) |
|  | 2021 | 24 (00ˑ38%) | 5420 (86ˑ11%) | 850 (13ˑ50%) |
|  | 2022 | 8 (00ˑ13%) | 5334 (87ˑ23%) | 773 (12ˑ64%) |
|  |  |  |  |  |
| Sample Size | Quartile 1 | 1812 (33ˑ08%) | 3339 (60ˑ95%) | 327 (5ˑ97%) |
|  | Quartile 2 | 2098 (38ˑ30%) | 3015 (55ˑ04%) | 365 (6ˑ66%) |
|  | Quartile 3 | 1540 (28ˑ12%) | 3446 (62ˑ92%) | 491 (8ˑ96%) |
|  | Quartile 4 | 1723 (31ˑ46%) | 3232 (59ˑ01%) | 522 (9ˑ53%) |
|  |  |  |  |  |
| Type of Sponsor | Government | 1514 (35ˑ10%) | 2409 (55ˑ85%) | 390 (9ˑ04%) |
|  | Industry | 544 (28ˑ22%) | 1335 (69ˑ24%) | 49 (2ˑ54%) |
|  | Private (hospital and college) | 834 (33ˑ83%) | 1407 (57ˑ08%) | 224 (9ˑ09%) |
|  | Research Institution | 1929 (36ˑ95%) | 2906 (55ˑ67%) | 385 (7ˑ38%) |
|  | Others | 2352 (29ˑ46%) | 4975 (62ˑ31%) | 657 (8ˑ23%) |
|  |  |  |  |  |
| PG Thesis | No | 2574 (32ˑ88%) | 4652 (59ˑ42%) | 603 (7ˑ70%) |
|  | Yes | 4593 (32ˑ66%) | 8366 (59ˑ50%) | 1102 (7ˑ84%) |
|  |  |  |  |  |
| No of countries | Multi country | 137 (32ˑ54%) | 269 (63ˑ90%) | 15 (3ˑ56%) |
|  | Single country (India) | 7036 (32ˑ74%) | 12763 (59ˑ39%) | 1690 (7ˑ86%) |
|  |  |  |  |  |
| Regulatory Approval | Applicable (Approved/Obtained, Awaited, No Objection Certificate, Notified) | 344 (30ˑ28%) | 768 (67ˑ61%) | 24 (2ˑ11%) |
|  | Not Applicable | 6829 (32ˑ87%) | 12264 (59ˑ04%) | 1681 (8ˑ09%) |
|  |  |  |  |  |
| Phase | Phase 3 (2/3,3,3/4) | 1271 (29ˑ59%) | 2673 (62ˑ24%) | 351 (8ˑ17%) |
|  | Others | 2482 (40ˑ14%) | 3326 (53ˑ79%) | 375 (6ˑ07%) |
|  | N/A | 3420 (29ˑ92%) | 7033 (61ˑ52%) | 979 (8ˑ56%) |
|  |  |  |  |  |
| Study Design | Single Arm | 1042 (31ˑ29%) | 2096 (62ˑ94%) | 192 (5ˑ77%) |
|  | Non randomized | 271 (37ˑ23%) | 413 (56ˑ73%) | 44 (6ˑ04%) |
|  | Randomized | 5089 (33ˑ13%) | 8938 (58ˑ18%) | 1335 (8ˑ69%) |
|  | Other | 771 (30ˑ96%) | 1585 (63ˑ65%) | 134 (5ˑ38%) |

Of the 21,910 included trial records, 7,173 (32ˑ74%) did not provide any information in the IPD sharing plan field in CTRI, 13,032 (59ˑ48%) did not intend to share IPD and only 1,705 (7ˑ78%) committed to sharing IPD.

**Figure 1 Year-wise breakdown of IPD sharing statements of CTRI records included in our analysis**


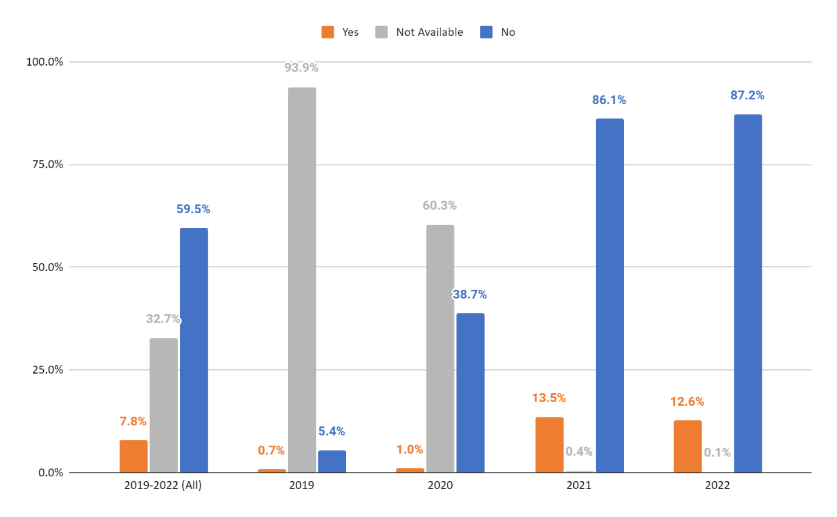


We carried out a sub-analysis of all 1,075 trials that had expressed an intent to share their IPD. Of these 649 (38ˑ13%) trials records intended to share the complete data, while the rest of the trials only planned to share the IPD for the results that were mentioned in the trial's publication. 459 (26ˑ98%) trial records would share their data with anyone, while 722 (42ˑ45%) specified that they'd only share IPD with researchers whose reanalysis plan was approved by an independent review committee. Email to the trialist was the most popular data-sharing mechanism, as specified by 1,101 (64ˑ73%) trials. Table 2 summarizes the sub-questions answered by the trialists that intended to share their IPD.

**Table 2 Mechanism and extent of data sharing by the 1,705 trials that intend to share IPD**

| Sub question No | Sub questions | Sub question answer options | N (%) |
| --- | --- | --- | --- |
| 1 | What data in particular will be shared? | All of the individual participant data collected during the trial, after de-identiﬁcation. | 649 (38ˑ13%) |
|  |  | Individual participant data that underlie the results reported in specific publication arising out of the trial, after de-identiﬁcation (text, tables, ﬁgures, and appendices). | 988 (58ˑ05%) |
|  |  | Other | 65 (3ˑ82%) |
| 2 | What additional supporting information will be shared? [Choose all that apply] | Study Protocol | 1495 (87ˑ89%) |
|  |  | Statistical Analysis Plan | 1192 (70ˑ08%) |
|  |  | Informed Consent Form | 1044 (61ˑ38%) |
|  |  | Clinical Study Report | 1059 (62ˑ26%) |
|  |  | Analytic Code | 325 (19ˑ11%) |
|  |  | None of the above | 43 (2ˑ53%) |
| 3 | Who will be able to view these files? | Anyone | 459 (26ˑ98%) |
|  |  | Researchers who provide a methodologically sound proposal. | 448 (26ˑ34%) |
|  |  | Researchers whose proposed use of the data has been approved by an independent review committee identiﬁed for this purpose. | 722 (42ˑ45%) |
|  |  | Other | 72 (4ˑ23%) |
| 4 | For what types of analyses will this data be available? | Any purpose | 449 (26ˑ40%) |
|  |  | To achieve aims in the approved proposal. | 805 (47ˑ33%) |
|  |  | For individual participant data meta-analysis. | 369 (21ˑ69%) |
|  |  | Other | 78 (4ˑ59%) |
| 5 | By what mechanism will data be made available? | Data are available indeﬁnitely at (Link to be included) | 68 (4ˑ00%) |
|  |  | Proposals should be directed to [email ID to be provided] | 1101 (64ˑ73%) |
|  |  | To gain access, data requestors will need to sign a data access agreement. Data are available for 5 years at a third-party website (Link to be included) | 15 (0ˑ88%) |
|  |  | Others | 517 (30ˑ39%) |
| 6 | For how long will this data be available? [Please provide tentative future dates taking into account trial completion dates and proposed timelines for manuscript preparation and publication] | Immediately following publication. No end dates. | 575 (33ˑ80%) |
|  |  | Beginning 3 months and ending 5 years following article publication | 534 (31ˑ39%) |
|  |  | Beginning 9 months and ending 36 months following article publication. | 330 (19ˑ40%) |
|  |  | Other | 262 (15ˑ40%) |
| 7 | Any URL or additional information regarding plan/policy for sharing IPD? (Please write NIL if there is no additional information) | Nil | 1616 (94ˑ78%) |
|  |  | Other | 89 (5ˑ22%) |

We also looked at the study characteristics of trials that did not plan to share their IPD, and found that trials that were industry-funded (2ˑ54%), required regulatory approval (2ˑ11%), or were multi-country studies (3ˑ56%) were less likely to share their IPD.

The interactive living dashboard is available at <https://www.georgeinstitute.org/intent-to-share-individual-participant-data-in-indian-trials-a-living-analysis-of-the-clinical>

This dashboard will be updated with newer CTRI records on a yearly basis.

# References

1. Borana, R., & Bhaumik, S. (2022). Compliance with International Committee of Medical Journal Editors policy on individual participant data sharing in clinical trial registries: An audit. Perspectives in Clinical Research, 13(4), 213
2. Taichman DB, Backus J, Baethge C, et al. Sharing Clinical Trial Data: A Proposal From the International Committee of Medical Journal Editors. *Ann Intern Med*. 2016;164(7):505
3. Tan AC, Askie LM, Hunter KE, Barba A, Simes RJ, Seidler AL. Data sharing—trialists' plans at registration, attitudes, barriers and facilitators: A cohort study and cross‐sectional survey. *Res Synth Methods*. 2021;12(5):641-657
4. Merson, L., Ndwandwe, D., Malinga, T., Paparella, G., Oneil, K., Karam, G., & Terry, R. F. (2022). Promotion of data sharing needs more than an emergency: An analysis of trends across clinical trials registered on the International Clinical Trials Registry Platform. *Wellcome open research*, *7*.
5. Statham, E. E., White, S. A., Sonwane, B., & Bierer, B. E. (2020). Primed to comply: individual participant data sharing statements on ClinicalTrials. gov. *PLoS One*, *15*(2), e0226143.
6. WHO Trial Registration Data Set (Version 1.3 - archived). Accessed 15 May, 2022. https://www.who.int/clinical-trials-registry-platform/network/who-data-set/archived/1-3
